# Glucagon regulates hepatic lipid metabolism via cAMP and Insig-2 signaling: implication for the pathogenesis of hypertriglyceridemia and hepatic steatosis

**DOI:** 10.1038/srep32246

**Published:** 2016-09-01

**Authors:** Hai Wang, Miaoyun Zhao, Neetu Sud, Patricia Christian, Jing Shen, Yongyan Song, Anjeza Pashaj, Kezhong Zhang, Timothy Carr, Qiaozhu Su

**Affiliations:** 1 The Department of Nutrition and Health Sciences, University of Nebraska-Lincoln, Lincoln, NE 68583-0806, USA; 2 Center for Molecular Medicine and Genetics, Wayne State University, School of Medicine, Detroit, MI 48201, USA

## Abstract

Insulin induced gene-2 (Insig-2) is an ER-resident protein that inhibits the activation of sterol regulatory element-binding proteins (SREBPs). However, cellular factors that regulate Insig-2 expression have not yet been identified. Here we reported that cyclic AMP-responsive element-binding protein H (CREBH) positively regulates mRNA and protein expression of a liver specific isoform of Insig-2, Insig-2a, which in turn hinders SREBP-1c activation and inhibits hepatic de novo lipogenesis. CREBH binds to the evolutionally conserved CRE-BP binding elements located in the enhancer region of Insig-2a and upregulates its mRNA and protein expression. Metabolic hormone glucagon and nutritional fasting activated CREBH, which upregulated expression of Insig-2a in hepatocytes and inhibited SREBP-1c activation. In contrast, genetic depletion of CREBH decreased Insig-2a expression, leading to the activation of SREBP-1c and its downstream lipogenic target enzymes. Compromising CREBH-Insig-2 signaling by siRNA interference against Insig-2 also disrupted the inhibitory effect of this signaling pathway on hepatic de novo triglyceride synthesis. These actions resulted in the accumulation of lipid droplets in hepatocytes and systemic hyperlipidemia. Our study identified CREBH as the first cellular protein that regulates Insig-2a expression. Glucagon activated the CREBH-Insig-2a signaling pathway to inhibit hepatic de novo lipogenesis and prevent the onset of hepatic steatosis and hypertriglyceridemia.

The development of hyperlipidemia is closely associated with the development of diabetes^1^^,2^. A family of b-zip transcription factors, the sterol regulatory element-binding proteins (SREBPs), is the master regulators of hepatic de novo lipogenesis, which target downstream genes involved in free fatty acid, triglyceride (TG) and cholesterol (CHOL) synthesis. Regulation of SREBP signaling is controlled by a cluster of ER membrane-bound proteins - insulin induced gene-1 (Insig-1) and gene-2 (Insig-2) and SREBP cleavage-activating protein (SCAP)^3^,^4^,^5^. Low sterol triggers a conformational change in the sterol-sensitive SCAP, causing the SCAP-SREBP complex to dissociate from the Insigs^3^,^6^. This allows the SCAP-SREBP complex to migrate to the Golgi apparatus, where the SREBPs are activated by proteolytic cleavages. These cleavages release the N-terminal portions of the SREBPs, which are the active forms that translocate to the nucleus and act as transcription factors to enhance transcription of genes required for de novo lipogenesis^6^. In the liver, SREBP-1c mainly regulates free fatty acid synthesis, while SREBP-2 governs cholesterol metabolism^5^.

Insig-1 and Insig-2 are two sister proteins that interact with and retain SREBPs in the ER, inhibiting their activation. Although Insig-2 is a close homolog of Insig-1, its physiological activity is regulated by mechanisms different from those that regulate Insig-1. Insulin induces mRNA expression of Insig-1 and activates SREBPs but inhibits Insig-2^5^. mRNA of Insig-2 consists of two isoforms, Insig-2a and -2b, which display tissue specific distribution. Insig-2a is predominantly expressed in the liver, whereas Insig-2b is ubiquitously expressed in other tissues and organs^5^,^7^. Although these two isoforms differ in the enhancer regions of their mRNA structures, with Insig-2a mRNA containing a non-coding exon-1 and an approximate intron that are missing in the mRNA of Insig-2b, both isoforms are eventually spliced to give the same mRNA that encodes identical proteins, Insig-2. Metabolic hormone, insulin, and metabolites, namely sterols, have been shown to regulate Insig-2a cellular abundance. However, little is known about the cellular protein(s) involved in conveying signaling from metabolic hormone to Insig-2 expression thus far.

Although the mechanism of how hepatic SREBP-1c is induced by insulin in the fed state is well-established, little is known about how hepatic SREBP-1c is suppressed during the fasting state. Glucagon, a metabolic hormone released from pancreatic α cells, is the principal regulatory hormone that counters the actions of insulin. Exogenous glucagon reduces liver TG content and prevents the development of fatty liver in dairy cows^8^,^9^, whereas reduced glucagon action is associated with the development of fatty liver^10^,^11^. Emerging evidence has further demonstrated that, in the fasted state, glucagon action is essential for multiple pathways regulating lipid homeostasis^12^,^13^,^14^. Its inhibitory effect on hepatic de novo lipogenesis was proposed to be mediated by the cAMP/protein kinase A pathways. However, the mechanism of action between glucagon/cAMP signaling and SREBP activation is not well defined. cAMP-responsive element-binding protein H (CREBH) is a recently identified transcription factor that is structurally related to the SREBPs^15^. Similar to Insig-2a, CREBH is selectively and highly expressed in the liver^16^. CREBH is synthesized as an ER-resident precursor protein and activated by S1P and S2P proteases in the Golgi apparatus in a mode similar to SREBP activation^15,^^17^. Activation of CREBH is induced by nutritional factors, such as fasting and free fatty acids, and suppressed by refeeding^18^,^19^,^20^. Genetic depletion of CREBH in mice induced fasting hypoglycemia and hypertriglyceridemia compared to wild type littermates^20^,^21^. Heterozygous nonsynonymous or insertional mutations in human CREBH (CREB3L3) caused severe hypertriglyceridemia in these individuals^21^. These evidences suggest that CREBH may play a negative regulatory role in hepatic de novo lipid synthesis. However, the intrinsic association between CREBH and SREBPs, particularly the link between these two lipid metabolic transcription factors and their impacts in regulating hepatic lipid synthesis, haven’t been reported. In this study, we identify CREBH as the first cellular molecule that regulates Insig-2a at the transcriptional level. We further demonstrated that the CREBH-Insig-2a pathway inhibits hepatic de novo lipogenesis during the nutritional fasting state and is regulated by glucagon. Specifically, CREBH suppressed hepatic de novo lipid synthesis by upregulating the mRNA and protein expressions of Insig-2a. Chromatin Immunoprecipitation (ChIP) assay demonstrated the functional association between CREBH and the CRE-BP binding elements within the enhancer region of the Insig-2a gene. Nutritional signaling, fasting, and metabolic hormone glucagon activated CREBH, which subsequently induced Insig-2a expression to inhibit SREBP-1c activation. In contrast, compromising CREBH activity through genetic depletion decreased hepatic abundance of Insig-2a. Depletion of Insig-2 by siRNA interference disrupted the inhibitory effect of CREBH-Insig-2a on hepatic lipid synthesis. These actions led to the activation of SREBP-1c and its downstream lipogenic target enzymes as well as the subsequent de novo lipid synthesis upon fasting, resulting in hepatic steatosis and systemic hyperlipidemia.

## Results

### Insig-2a mRNA and protein expression are regulated by CREBH in response to fasting and refeeding

**Figure 1:**
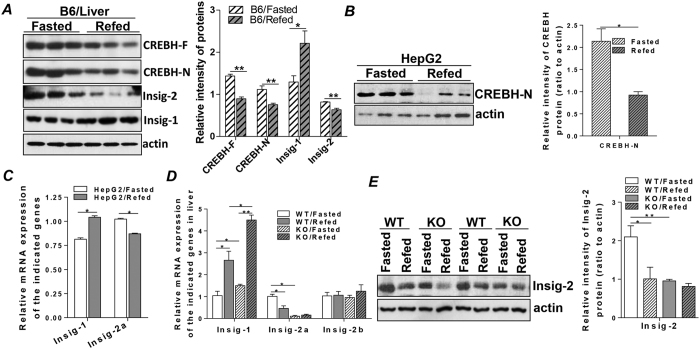
Insig-2a mRNA and protein expression are regulated by CREBH in response to fasting and refeeding. C57/B6 WT mice were divided into two groups (n = 6/group) and subjected to the fasting and refeeding protocol as described in the Methods. Livers were then collected and used in the following analyses. (**A**) Immunoblot analysis of full length-CREBH (CREBH-F), active form of CREBH (CREBH-N), Insig-1 and Insig-2 in the livers of the fasted and refed mice. (**B**) HepG2 cells (1 × 10^6^) were starved without FBS in low glucose DMEM (glucose 1 g/ml) for 18 hours and refed by adding 5% FBS to the high glucose DMEM (4.5 g/ml) for 8 hours, after which whole cell lysates were subjected to immunoblot analysis for the active form of CREBH, CREBH-N. (**C**) Relative mRNA expression of Insig-1 and Insig-2a detected by qRT-PCR in HepG2 cells as prepared in (B). (**D**) CREBH-KO and control littermates (WT) were divided into fasted and refed groups (n = 6/group) and subjected to the fasting and refeeding protocol as described in the Methods. Livers were then harvested, RNA was extracted, and relative mRNA levels of Insig-1, Insig-2a and Insig-2b were analyzed by qRT-PCR. (**E**) Immunoblot analysis of Insig-2 in the liver homogenates from mice prepared as in (D). KO represents CREBH-KO. For animal studies, results are shown as mean ± SEM. For *in vitro* studies, data represent three experiments. *P < 0.05, **P < 0.01 versus controls.

Previously, mRNA expression of Insig-2a was reported to be induced by fasting and suppressed by refeeding by Drs. Goldstein and Brown’s group^22^. Subsequently, Danno *et al*. showed that CREBH is elevated during nutrition depletion^18^. To investigate whether there is intrinsic association between these two ER-resident and liver-expressed proteins, we subjected two groups of mice (n = 6/group) to a fasting and refeeding protocol, as described in the Methods. After determining the mRNA levels of CREBH and Insig-2a in the livers of the mice by quantitative real-time PCR, we found that both CREBH and Insig-2a mRNA levels concomitantly rose at the fasting state and fell upon refeeding (Supplementary Fig. S1A). In contrast, mRNA level of Insig-2b, the other isoform of Insig-2, which is expressed ubiquitously, was not affected by the nutrient switch (Supplementary Fig. S1A). Consistent with a previous report^22^, Insig-1 mRNA was significantly reduced or induced by fasting and refeeding, respectively (Supplementary Fig. S1A). Immunoblot analysis further showed that activation of CREBH induced by fasting, shown through the increased expression of the N-terminus of CREBH (CREBH-N), was accompanied by marked upregulation of Insig-2 protein mass, compared to the refed state (Fig. 1A). Reciprocally, protein level of Insig-1 was significantly lower during the fasting state but rose upon refeeding (Fig. 1A). *In vitro*, mimicking the fasted state by depleting serum from the culture medium (glucose 1 g/ml) of a human hepatoma cell line, HepG2 cells, for 18 hours activated CREBH, indicated by the increased abundance of active CREBH, N-terminal CREBH (CREBH-N) (Fig. 1B). Fasting further stimulated expression of Insig-2a mRNA (Fig. 1C). Refeeding the fasted HepG2 cells with complete medium (FBS 5%; glucose 4.5 g/ml) suppressed CREBH activation and inhibited Insig-2a mRNA expression (Fig. 1B,C). In contrast, refeeding enhanced Insig-1 mRNA expression in HepG2 cells (Fig. 1C). These results suggested the potential regulatory effect of CREBH on Insig-2a.

To further explore the intrinsic physiological association between CREBH and Insig-2a, Insig mRNAs were measured in the liver tissues from CrebH knockout mice (CREBH-KO) and their wild type littermates (WT) under chow-diet conditions. We reasoned that if the mRNA abundance of Insig-2a is regulated by CREBH, depletion of CREBH should be able to prevent the responsive changes of Insig-2a mRNA upon stimulation with fasting and refeeding. Indeed, we found that compared to the WT controls, Insig-2a mRNAs in the livers of the KO mice were expressed at a significantly lower level, which failed to respond to the stimulation of fasting and refeeding (Fig. 1D). In contrast, Insig-1 mRNAs in the KO mice were significantly increased compared to that of the WT controls. This may suggest a compensatory feedback response on this Insig sister protein. mRNAs of Insig-2b remained unchanged in both WT and KO mice (Fig. 1D). Modification of Insig-1 and Insig-2a mRNA transcripts by CREBH depletion were further reflected on the protein expression of hepatic Insig-1 and -2. Immunoblot analysis showed that Insig-1 protein expression was upregulated at both fasted and refed states in the KO mice, whereas Insig-2 protein mass was significantly lower compared to the WT at the fasted state, where CREBH was activated (Fig. 1E and Supplementary Fig. S1B). Expression of Insig-1 mRNA has been shown to be upregulated by insulin^5^, we thus measured the plasma insulin contents and found that plasma insulin levels were significantly elevated in the fasted-KO mice compared to the fasted-WT mice (Supplementary Fig. S1C). Refeeding stimulated insulin secretion in both WT and KO mice but a higher insulin level was noted in the refed-KO compared to refed-WT mice (Supplementary Fig. S1C). The regulatory effect of CREBH occurred specifically in the liver as we failed to detect statistically significant changes in Insig-2b mRNAs in the white adipose tissues of both WT and KO mice, while Insig-2a mRNA was undetectable in this tissue (Supplementary Fig. S1D). This observation is in line with the physiological distribution of both CREBH and Insig-2a, which are selectively and highly expressed in the liver^5^,^16^. Together, these results indicated that activation of CREBH specifically enhances mRNA and protein expression of Insig-2a but not Insig-2b or Insig-1 in response to nutritional starvation.

### Impairment of CREBH and Insig-2a signaling augments hepatic de novo lipogenesis in the fasting state in CREBH-KO mice

**Figure 2:**
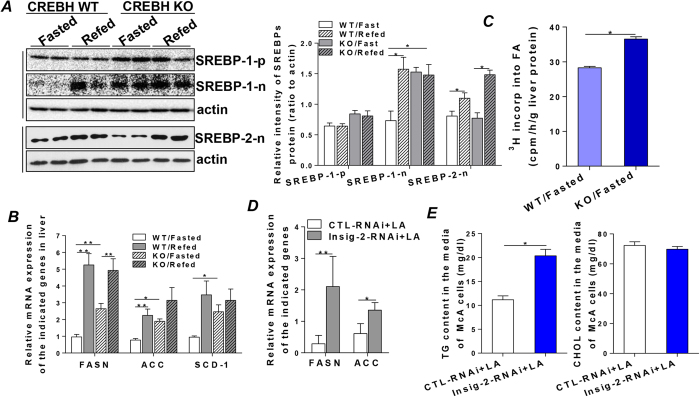
Impairment of CREBH and Insig-2a signaling augments hepatic de novo lipogenesis at the fasting state in CREBH-KO mice. Liver tissues that were harvested from WT and CREBH-KO mice subjected to the fasting and refeeding protocol as described in the Methods were used to prepare total RNA and tissue homogenates for the following analyses. (**A**) Protein mass of the precursor, as well as the active forms of SREBP-1 (SREBP-1-n) and SREBP-2 (SREBP-2-n) were measured by immunoblot analysis. Immunoblots were quantified by densitometry and were normalized to β-actin. (**B**) Relative mRNA expression of SREBP-1c target genes, FASN, ACC, and SCD-1 were determined by qRT-PCR. (**C**) Hepatic lipogenesis was determined by intraperitoneal injection of ^3^H_2_O into fasted-WT and fasted-KO mice (n = 3/group). Incorporation of ^3^H_2_O into newly synthesized fatty acids was determined as described in the Methods. (**D**) McA cells were transfected with siRNA against Insig-2 or control siRNA for 36 hours, cells were then treated with glucose (6mM) and LA (200 μM) for additional 36 hours. Cells were used to prepare total RNA and relative mRNA expression of FASN and ACC were determined by qRT-PCR. (**E**) Secreted TG and CHOL in the culture media of McA cells treated as in (D) were extracted and determined as described in the Methods. KO represents CREBH-KO. For animal studies, results are shown as mean ± SEM. n = 3–12/group. For *in vitro* studies, data represent three experiments. *P < 0.05, **P < 0.01 versus controls.

**Figure 3:**
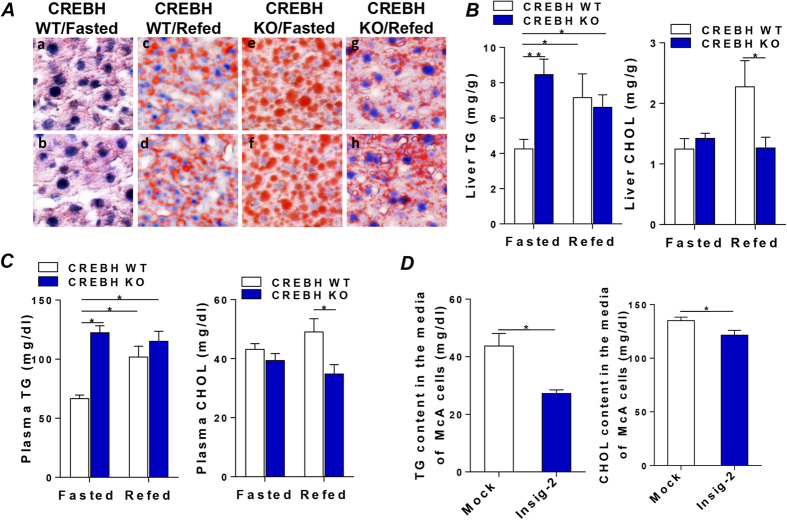
Defective CREBH and Insig-2a signaling induces hepatic steatosis and hyperlipidemia in the fasting state. (**A**) Representative photographs demonstrating the appearance of livers from WT and CREBH-KO mice after being subjected to the fasting and refeeding protocol as described in the Methods. The livers were harvested and frozen liver sections were subjected to staining with the lipid-specific oil red-O dye to positively reveal lipid droplets; a,b: WT/fasted; c,d: WT/refed; e,f: CREBH-KO/fasted; g,h: CREBH-KO/refed. (**B**) Liver TG and CHOL contents from mice as in (A) were determined by procedures detailed in the Methods. (**C**) Plasma TG and CHOL from CREBH-KO and their WT littermates after being subjected to the fasting and refeeding procedure described in the Methods (n = 16/group). (**D**) TG and CHOL contents in the culture media of McA cells transfected with a mock control vector or Insig-2 for 48 hours were determined by procedure described in the Method. For *in vitro* studies, results are shown as means ± SD for two experiments that were performed in triplicate. For animal study, results are shown as mean ± SEM. *P < 0.05, **P < 0.01 versus controls.

To determine the regulatory role of the CREBH-Insig-2 axis on hepatic lipid metabolism, we first investigated whether CREBH regulates SREBPs on the transcriptional level. Overexpression of a control vector or a cDNA encompassing CREBH wild type (CREBH WT) in McA-RH7777 (McA) cells, a rat hepatoma cell line, was unable to stimulate mRNA transcription of SREBP-1c and SREBP-2 (Supplementary Fig. S2A), indicating that CREBH is unlikely to regulate SREBPs at the transcriptional level. Since Insigs are the key proteins that sequester the SCAP-SREBP complex in the ER and inhibit SREBP activation^5^,^22^, we thus investigated the consequence of the defective CREBH and Insig-2a activities on hepatic lipid metabolism by determining the activation of SREBPs and their target enzymes involved in lipid synthesis during the fasting and refeeding states. In the fasting state, although the precursor of SREBP-1c was comparable between WT and KO mice, the abundance of the active SREBP-1c (SREBP-1c-n) was significantly elevated in the fasted-KO mice compared to the fasted-WT littermates (Fig. 2A), suggesting a post-translational activation of SREBP-1c in the CREBH-KO mice during fasting. More importantly, activation of SREBP-1c in the fasted-KO mice stimulated de novo lipid synthesis signaling in hepatocytes, indicated by the significantly higher mRNAs of the lipogenic enzymes that are involved in free fatty acid synthesis, including *fasn, acc* and *scd-1*, in the livers of the fasted-KO mice relative to those in the fasted-WT mice (Fig. 2B). Refeeding stimulated activation of SREBP-1c and SREBP-2 in both groups of mice (Fig. 2A), which in turn augmented hepatic de novo lipogenesis signaling by significantly enhancing the mRNA transcription of the lipogenic enzymes, *fasn, acc* and *scd-1*, as well as *hmgcs, hmgcr* and *ldl*-*receptor* (Fig. 2B and Supplementary Fig. S2B). The biological consequence of hyperactivation of SREBP-1c was further determined by measuring hepatic de novo lipogenesis using ^3^H_2_O as tracer by the procedure described in ref. 23. Figure 2C shows that depletion of CREBH significantly increased incorporation of ^3^H_2_O into fatty acids in the livers of the fasted-KO mice, suggesting an increased rate of hepatic de novo lipid synthesis (Fig. 3C). Previously, we have showed that a chemical compound, lipoic acid (LA), activates CREBH *in vitro* and *in vivo*^24^. To further determine the specific role of the CREBH-Insig-2a axis in regulating hepatic lipid metabolism, we used siRNA against Insig-2 to silence the endogenous Insig-2 in McA cells and activated CREBH by LA. We reasoned that, if CREBH-Insig-2 signaling is critical in regulating hepatic de novo fatty acid synthesis, silencing Insig-2 would disrupt the inhibitory effect of CREBH-Insig-2 signaling on glucose-induced lipogenesis. Indeed, after delivering a control siRNA or a siRNA specifically against Insig-2 into McA cells for 36 hours followed by treating the cells with glucose (6 mM) and LA (200 nM) for 36 hours, we found that mRNAs of *fasn* and *acc* were significantly higher in the Insig-2 siRNA-transfected cells compared to the control-siRNA transfected cells (Fig. 2D). Determination of the lipids secreted by McA cells revealed that TG content was significantly higher in the Insig-2 siRNA-transfected cells compared to the controls while CHOL content had no significant change (Fig. 2E). Taken together, these results strongly demonstrated that the CREBH-Insig-2a signaling pathway inhibits hepatic de novo lipid synthesis at the interface of the fasting and refeeding.

### Defective CREBH and Insig-2a signaling induces hepatic steatosis and hyperlipidemia in the fasting state

To further assess the outcome of the hyperactivation of hepatic lipogenic pathways, we analyzed hepatic lipid contents with oil red O staining. Figure 3A shows the marked accumulation of lipid droplets in the livers of the fasted-KO mice compared to the fasted-WT mice (Fig. 3A, a, b compared with e, f). Refeeding resulted in accumulation of lipid droplets in the livers of both refed-WT and refed-KO mice (Fig. 3A, c, d and g, h). Quantification of the hepatic lipid contents was done by extracting and determining lipid contents in the mouse liver tissues using the protocol described in the Methods. Consistent with the oil red O stainingresults, fasting resulted in significant accumulation of TG in the KO liver compared to the WT (Fig. 3B). Refeeding raised hepatic TG in the WT mice but failed to further elevate TG in the KO mice (Fig. 3B). Depletion of CREBH did not significantly alter CHOL contents in the livers of either WT or KO mice at the fasting state (Fig. 3B). In contrast, refeeding significantly raised CHOL contents in livers of WT but not the KO mice (Fig. 3B). Upon further examining the plasma lipid contents, we found that plasma TG was significantly elevated in the fasted-KO, refed-WT and refed-KO mice compared to fasted-WT (Fig. 3C). Depletion of CREBH did not significantly alter plasma CHOL level in the fasted-KO mice compared to the fasted-WT (Fig. 3C). Refeeding markedly elevated plasma CHOL levels in WT but not in the KO mice (Fig. 3C) which was consistent with the liver CHOL contents (Fig. 3B). At a glance, this result is at odds with the hyperactivation of HMG-CoA reductase and HMG-CoA synthase (Supplementary Fig. S2B). However, it has been reported that binding of Insig-1 to the sterol-sensing domain of HMG-CoA reductase accelerates the degradation of this rate-limiting enzyme in cholesterol biosynthesis^25^. Because mRNA and protein levels of Insig-1 is significantly induced in the KO mice (Fig. 1D and Supplementary Fig. S1), whether or not the decrease of plasma CHOL in the KO mice is caused by the aberrant elevated level of Insig-1 requires further investigation. To investigate whether Insig-2 directly regulates hepatic VLDL-apoB synthesis, McA cells were transfected with a mock empty vector or a cDNA encompassing Insig-2 for 48 hours. Upon examining the secreted VLDL-TG and VLDL-apoB100 (apoB), a key structural apolipoprotein in the VLDL particles, in the culture media, we found that VLDL-TG was significantly lower in the Insig-2 transfected-cell media compared to the mock transfected cell media, whereas VLDL-apoB was comparable between these two treatments (Fig. 3D and Supplementary Fig. S3). This result suggests that reduced VLDL-TG secretion in the Insig-2 transfected-cells was more likely a consequence of lowered lipid substrates availablity caused by the reduction of hepatic de novo lipid synthesis rather than the suppression of apoB expression. Taken together, these data demonstrated that the CREBH-Insig-2a signaling pathway is critical for maintaining hepatic lipid homeostasis. Compromising the action of CREBH-Insig-2a signaling induces hepatic steatosis and systemic hyperlipidemia in the fasting state.

### Insig-2 is a target gene of CREBH which functionally interacts with the CREB-biding elements within the promoter of Insig-2

**Figure 4:**
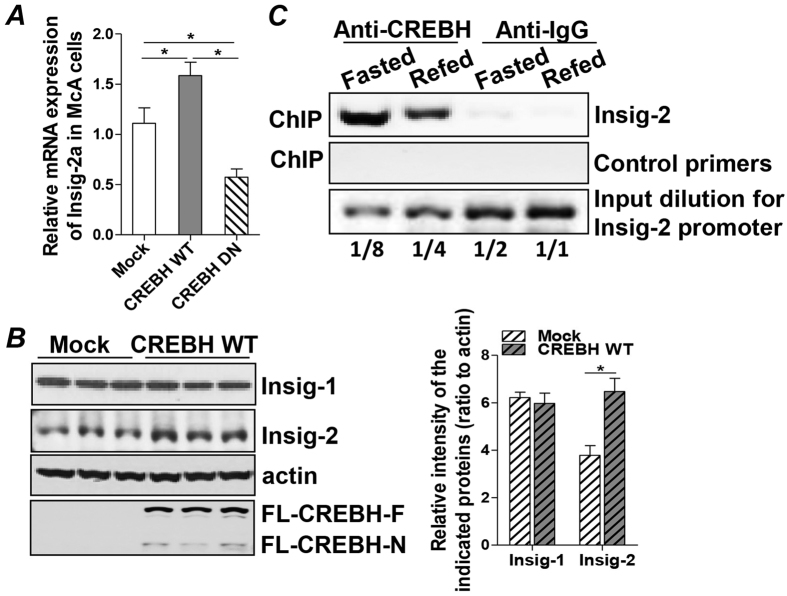
Insig-2 is a target gene of CREBH which functionally interacts with the CREB-biding elements within the promoter of Insig-2. (**A,B**) McA cells (1 × 10^6^) were transfected with 1.5  μg of an empty vector (Mock), 1.5  μg of a pCMV7.1 plasmid containing 3Flag-CREBH WT, or dominant negative CREBH (DN) cDNA. After 48 hours, transfected cells were harvested for total RNA and whole cell lysate preparation. (**A**) Relative Insig-2a mRNA expression detected by qRT-PCR and (**B**) Insig-1 and Insig-2 protein mass measured by immunoblot analysis. Expression of full length-CREBH and CREBH-N are shown in the bottom panel. (**C**) ChIP assay of Insig-2 gene promoter in mouse liver tissues. Briefly, after cross-linking chromatin DNA to the interacting proteins, specific immunoprecipitation with anti-CREBH antibody or a pre-immune-IgG as a negative control was performed as described in Methods. PCR products of the Insig-2 promoter were analyzed after PCR amplification (36 cycles) by using a pair of primers flanking the CRE-BP binding elements (upper panel). PCR products using a pair of negative control primers are shown in the middle panel. The lower panel of the figure shows verification of the quantitative aspect of the PCR amplification using serial dilutions of the input. Data represent three experiments. *P < 0.05 versus controls.

To further delineate the regulatory mechanism of CREBH on Insig-2a, we expressed a mock empty vector, a cDNA encompassing CREBH WT, or a dominant negative form of CREBH (CREBH DN) in McA cells. Analyzing the mRNA expression of Insig-2a, we found that forced expression of CREBH WT or CREBH DN either significantly stimulated or suppressed mRNA transcription of Insig-2a in McA cells, respectively (Fig. 4A). Protein mass of Insig-2 but not Insig-1 was also markedly induced in the presence of Flag-CREBH-WT compared to the mock transfection (Fig. 4B). These results indicated that CREBH positively regulates Insig-2a expression on the transcriptional and translational levels.

To further investigate the potential functional association between CREBH and the Insig-2 gene promoter, we analyzed the promoters and transcriptional enhancer regions of Insig-2 in three different species - human, rat and mouse - and identified two potential CRE-BP (−62) and CREB(+99) binding elements that are located proximally to the non-coding exon-1 of human Insig-2a (Supplementary Fig. S4A). More importantly, these two binding motifs and the gene sequences flanking these two binding sites are evolutionally conserved among human, rat and mouse, which may indicate their biological significances in regulating cellular activities (Supplementary Fig. S4A). To determine whether CREBH is physically associated with these binding sites, a chromatin immunoprecipitation (ChIP) assay was performed using specific anti-CREBH antibody to immunoprecipitate fragments of genomic DNA from the liver homogenates from fasted and refed mice. The result given in Fig. 4C shows the PCR amplification of a specific DNA fragment that encompassed the binding sites (Fig. 4C, upper panel). Activation of CREBH by fasting induced stronger interaction of CREBH with Insig-2 promoter in the liver of the fasted mice than in the refed mice (Fig. 4C, upper panel). The amplification of this fragment was undetectable in the negative control samples utilizing normal rabbit control IgG for immunoprecipitation (Fig. 4C, upper panel), although this fragment was present in the whole cell lysates (Fig. 4C, lower panel). A negative control primer pair was unable to amplify the DNA fragment immunoprecipitated by anti-CREBH antibody or control IgG (Fig. 4C, middle panel). The interaction of CREBH with the CRE-BP binding motifs within the Insig-2a promoter was further demonstrated in another rodent species, rat, by a ChIP assay as well (Supplementary Fig. S4B). Taken together, these data demonstrated that CREBH functionally associates with the CRE-BP binding elements in the enhancer region of the Insig-2 gene and positively regulates Insig-2 mRNA transcription.

### Glucagon inhibits hepatic lipid synthesis via the mediation of CREBH and Insig-2 signaling

**Figure 5:**
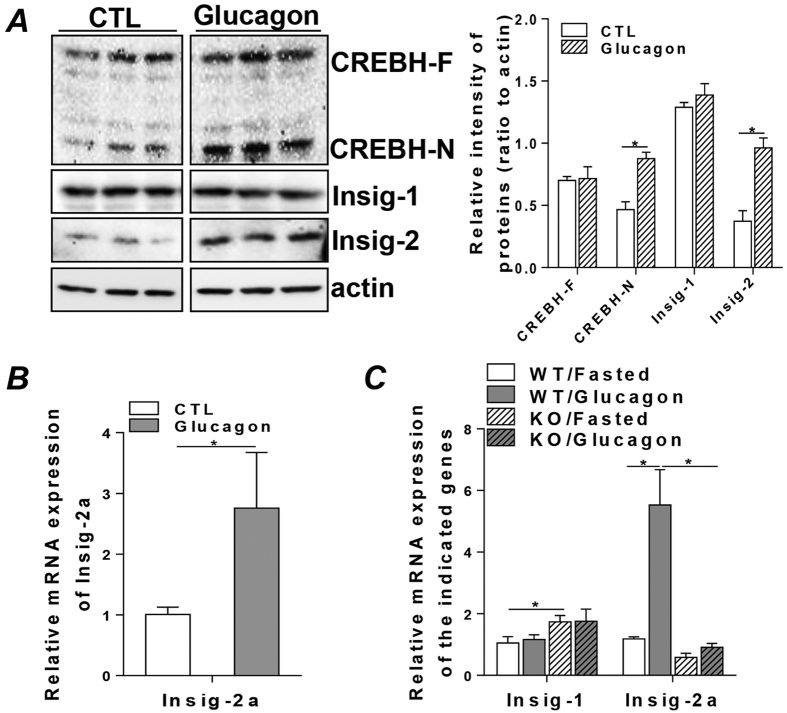
Glucagon inhibits hepatic lipid synthesis via the mediation of CREBH and Insig-2 signaling. (**A**,**B**) HepG2 cells (1 × 10^6^) were cultured without FBS for 12 hours, and then untreated or treated with 2 ng/ml glucagon for 24 hours. Cells were then harvested for total RNA and whole cell lysate preparation. (**A**) Protein mass of CREBH-F, CREBH-N, Insig-1 and Insig-2 were determined by immunoblot analysis (lanes were run on the same gel but noncontiguous, full images are shown in Supplementary Fig. 6). (**B**) Relative mRNA levels of Insig-2a by qRT-PCR in the glucagon treated HepG2 cells. (**C**) Four groups of WT or CREBH-KO (KO) mice were untreated or treated with glucagon (30 μg/kg) for 4 hours as detailed in the Methods. Total RNA was extracted from liver tissues and used to determine the mRNA expression of Insig-1 and Insig-2a by qRT-PCR. For animal studies, results are shown as mean ± SEM. n = 5–6/group. For *in vitro* studies, results are shown as means ± SD for two experiments that were performed in triplicate. *P < 0.05 versus controls.

Glucagon is a major metabolic hormone secreted during the fasted state that counters insulin signaling and promotes gluconeogenesis and glycogenolysis to maintain normal glycemia in the fasted state^5^,^12^. This action is mediated through the activation of a Gs protein, which leads to the stimulation of adenylate cyclase activity, cAMP production, and CREB activation^5^,^12^. To investigate whether glucagon alone is sufficient to activate CREBH and induce Insig-2a expression, we treated HepG2 cells with glucagon at a concentration of 2 ng/ml for 24 hours. Incubation with glucagon induced activation of CREBH in HepG2 cells, as evidenced by the elevated levels of the active form of CREBH (CREBH-N) (Fig. 5A, upper panel). The activation of CREBH in the glucagon-treated cells was accompanied by increased mRNA and protein expression of Insig-2a (Fig. 5A lower panel and B) but not Insig-1 (Fig. 5A, middle panel), suggesting that glucagon may be involved in the activation of CREBH and Insig-2a during the fasted sate. To further determine the specific role of CREBH in mediating the activation of Insig-2a by glucagon *in vivo*, we treated the WT and KO mice with glucagon (30 μg/kg) for a 4 hour time course as described in the Methods. Treatment with glucagon induced about 5-fold increase in Insig-2a mRNA expression in the WT mice but not in the KO mice (Fig. 5C). Glucagon treatment also induced marked reduction of plasma VLDL-TG, indicating the inhibitory effect of glucagon on VLDL secretion (Supplementary Fig. S5A). mRNA expression of apoC4, a direct target gene of CREBH^26^, was also determined in this experiment to serve as a positive indicator of CREBH activation. As shown in Supplementary Fig. 5B, mRNA of apoC4 but not apoC2 or apoA5 was raised about 6 fold upon glucagon treatment compared to the untreated mice (SupplementaryFig. S5B). Expression of Insig-1 mRNA was not significantly affected by glucagon treatment (Fig. 5C). These data strongly support that Insig-2 is a target gene of CREBH and glucagon activates the CREBH-Insig-2 axis to regulate hepatic lipid metabolism.

## Discussion

**Figure 6:**
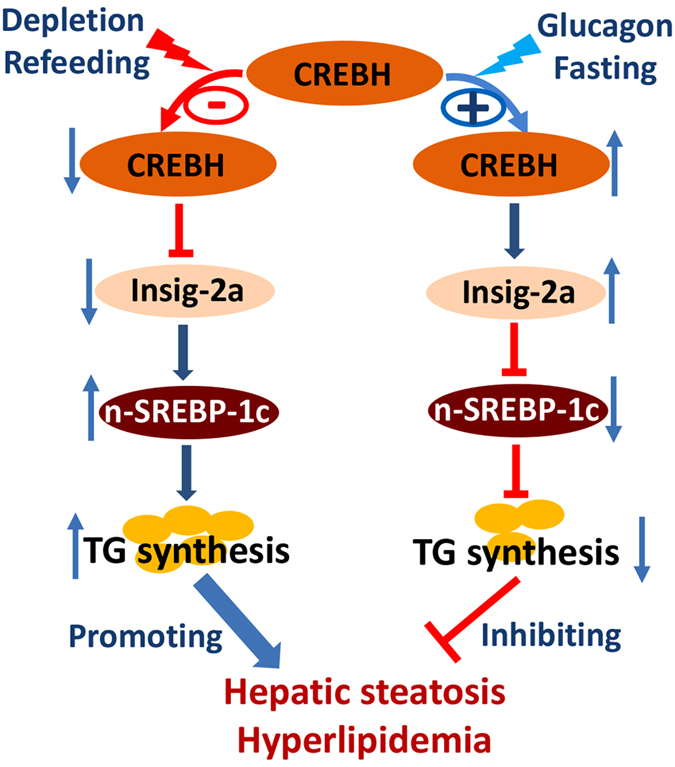
A schematic depicts the regulatory impact of the CREBH–Insig-2a signaling pathway on hepatic lipid metabolism. In hepatocytes, activation of CREBH by glucagon or fasting enhances expression of Insig-2a which subsequently sequesters and retains SREBP-1c in the ER, inhibits its activation and the downstream cascade of hepatic de novo lipid synthesis, leading to the improvement of hepatic steatosis and hyperlipidemia. In contrast, suppression of CREBH by genetic depletion or refeeding reduces expression of Insig-2a which results in the aberrant activation of SREBP-1c and de novo lipogenesis, leading to the accumulation of lipids in the hepatocytes and systemic hyperlipidemia.

The present study unveils a novel signaling pathway in hepatic lipid metabolism at the nutrient transition state between fasting and refeeding, which is regulated by glucagon (Fig. 6). This novel pathway involves CREBH and Insig-2a, two proteins that are located proximally to each other on the ER membrane and that are expressed tissue-specifically, mainly in hepatocytes. We demonstrated that activation of CREBH during the fasting state or by glucagon suppresses hepatic de novo lipid synthesis by enhancing the mRNA and protein expression of Insig-2a to hinder the activation of SREBP-1c. This regulatory signaling exerts its greatest effect on hepatic de novo triglyceride synthesis at the fasting state where glucagon is one of the major metabolic hormones. Genetic depletion of CREBH in mice reduces Insig-2a expression, leading to the activation of SREBP-1c and the subsequent activation of its lipogenic target enzymes. Blunting CREBH-Insig-2 signaling by siRNA interference against Insig-2 disrupted the inhibitory effect of this signaling pathway on hepatic de novo lipid synthesis, leading to aberrant accumulation of lipid droplets in hepatocytes and systemic hypertriglyceridemia during the nutritional fasting state (Fig. 6).

Hepatic lipogenesis, in which SREBPs and their target lipogenic enzymes are essential components, is regulated by nutritional status^27^. Insig-1, Insig-2 and SCAP are proteins that are associated with and regulate activation of SREBPs^28^,^29^. The anti-lipogenic actions of Insig-1 and Insig-2 have been reported in studies whereoverexpression of recombinant Insig-1 or -2 cDNA in Zucker diabetic fatty (ZDF) (fa/fa) rats substantially attenuated hepatic steatosis and hyperlipidemia^30^,^31^. Human studies further revealed that Insig-2 promoter polymorphism is an obesity-predisposing genotype that is present in 10% of obese individuals^32^. While these studies suggest that perturbation of Insig-2 protein abundance contributes to the development of obesity, hepatic steatosis andhyperlipidemia, little is known about the underlying mechanism and the cellular regulator(s) of Insig-2. In this study, we identified the first cellular protein, the transcription factor CREBH, as a positive regulator of Insig-2. Furthermore, glucagon activates the CREBH-Insig-2a signaling pathway in the liver. CREBH exerts it regulatory effect on Insig-2 through functionally associated with the CRE-BP binding sites at the enhancing region of Insig-2 and positively regulated the transcription and translation of Insig-2(a). Increased abundance of Insig-2 inhibits activation of SREBP-1c in the liver. Of note, we further noticed that although depletion of CREBH reduces Insig-2a, the expressions of Insig-1 mRNA and protein are significantly above that of the WT controls at both fasted and refed states. We further noticed that the elevated Insig-1 in the KO mice may be associated with the increased insulin secretion in KO mice as plasma insulin contents were significantly higher in these mice. Increased Insig-1 in this context could be a metabolic response in an attempt to compensate for the deficiency of Insig-2 in the KO mice and to hinder the aberrant activation of SREBP-1c. However, despite the compensatory increase of Insig-1, de novo lipid synthesis rate in hepatocytes of KO mice was still significantly higher compared to the WT mice. This finding may indicate that Insig-2a plays a more significant role in regulating lipid metabolism. Moreover, we further noticed that, although the protein abundance of nuclear SREBP-1c were comparable between fasted-KO and refed-KO mice, mRNA expression of the lipogenic genes, *fasn* and *acc*, in the refed-KO mice were significantly higher in the refed-KO than that in the fasted-KO mice. The mechanism for this observation is currently unknown. One possibility is the participation of other transcription factor(s) that directly induce mRNA transcription of *fasn* and *acc*. For instance, the transcription factor, liver X receptor (LXR), is able to directly interact with the *fasn* gene promoter and induce its transcription^33^. It is also possible that insulin induced by refeeding modulates the phosphorylation status of nuclear SREBP-1c and enhances its association with the promoter of its target gene to increase transcriptional efficiency^34^,^35^,^36^. More research is definitely warranted to delineate the mechanism for this phenotype.

Insulin has been shown to enhance the turnover rate of Insig-2 mRNA, which causes depletion of Insig-2, and the subsequent activation of lipogenic signaling. Glucagon stimulates cAMP formation and cAMP response element binding protein activation and inhibits lipogenic gene expression via the mediation of PPARγ^37^. In this study, for the first time, we demonstrated that glucagon upregulates Insig-2 mRNA and protein expression through the mediation of a new member of the CREB family, CREBH, which in turn suppressed hepatic lipid synthesis. Depletion of CREBH abolished the response of Insig-2 to glucagon treatment. Silencing the expression of Insig-2 by siRNA interference disrupted the inhibitory effect of CREBH-Insig-2a signaling on hepatic de novo lipid synthesis. Taken together, these findings may have clinical implications for therapeutic strategies to use glucagon to activate the cAMP signaling molecule CREBH and increase hepatic Insig-2a abundance in the treatment of hepatic steatosis and hyperlipidemia.

## Methods

### Cell culture and treatments

Human and rat hepatoma cell lines, HepG2 (ATCC) and McA (ATCC) cells, were maintained in DMEM containing 5% or 10% FBS at 37 °C, 5% CO2. Glucagon (EMD4 Biosciences, Millipore, MA) was used at final concentrations of 2 ng/ml for 24 hours.

### Animal protocols

All animal experiments were approved by the University of Nebraska-Lincoln Institutional Animal Care and Use Committee and were carried out under the institutional guidelines for ethical animal use. Mice used in this study were 10–14 weeks old. WT (C57BL/6J) mice were purchased from Jackson Laboratory (Bar Harbor, Maine, USA). CREBH knockout mice with exons 4–7 of the CrebH gene deleted were previously described^16^. Animals were housed on alternating 12-hour light and dark cycles with free access to food and water and placed on a chow diet (Dyets Inc., USA). After a week of acclimatization, mice were subjected to fasting for 12 hour or fasting for 12 hour followed by refeeding for 6 hour, n = 3–16/ per group. For glucagon treatment, mice were fasted for 8 hours followed by IP injection with glucagon (30 μg/kg body weight in 10% gelatine) or 10% gelatine alone. This treatment involved a total of 6 injections over a 4-hour period. Mice were then administrated a final dose (7^th^ dose) of glucagon via portal vein two minutes before being euthanized. Plasmas and liver tissues were collected and livers were homogenized in solubilization buffer as previously described^38^.

### Immunoblot analyses

Immunoblotting was performed as previously described^39^. The following antibodies were used in this study: anti-CREBH, anti-Insig-1 and anti-Insig-2 (Santa Cruz, USA); anti-SREBP-1 and anti-SREBP-2 (Novus, USA); anti-Flag (Cell signaling, USA). All antibodies were used at a final concentration of 0.1–1 μg/ml. After incubation with the appropriate horseradish peroxidase-conjugated anti-mouse or anti-rabbit IgG secondary antibody (1:5000 dilution; GE Healthcare UK), proteins were visualized by enhanced chemiluminescence (ECL) according to the manufacturer’s instructions (Amersham Biosciences, Pittsburgh, PA, USA).

### Plasmids and transfection

Plasmid pFlag-CREBH WT and pFlag-CREBH-DN were kindly provided by Dr. Randal J. Kaufman (Howard Hughes Medical Institute, University of Michigan Medical Center, Ann Arbor, Michigan, USA) and were previously described^15^. pCMV-Insig-2-Myc encoding human Insig-2 was kindly provided by Dr. Jin Ye (University of Texas Southwestern Medical Center, Dallas, Texas, USA) and was previously described^40^. For cell transfection, 1.5 μg of plasmid DNAs were transfected into McA cells as previously described^38^. For siRNA transfection, 100 nmol of siRNA against rat Insig-2 or control siRNA were transfected into McA cells as previous described^38^. 36 hours after the siRNA transfection, cells were treated with glucose (6 mM) and lipoic acid (LA) (200 μM) for additional 36 hours as described in ref. 24. Cells were collected for total RNA extraction and cell media were used for lipid extraction.

### Chromatin Immunoprecipitation assay

4 × 10^7^ cells were subjected to ChIP assay using Simple ChIP TM Enzymatic Chromatin IP Kit (Cell Signaling, USA) and anti-CREBH polyclonal antibodies or normal-rabbit IgG (Santa Cruz, USA). The assay was performed according to the manufacturer’s instructions with minor modification to the PCR repeating cycles (36 cycles). The PCR primer sets used for analysis of the mouse Insig-2 promoter were ACACCGGAAGTCCTTTTGCC (forward) and AGCTCCTCTTCCCAAAAGCC (reverse), which flank the CRE-BP binding elements in the mouse Insig-2 promoter.

### Measurement of hepatic de novo lipogenesis

The rate of hepatic de novo lipogenesis was determined by measuring the amount of newly synthesized fatty acids present in the liver 1 hour after intraperitoneal injection of 2 mCi/mouse of ^3^H_2_O (PerkinElmer) as described^23^. ^3^H-labeled fatty acids were isolated by saponification of liver samples in KOH. After extraction of nonsaponifiable lipids, and acidification with H_2_SO_4_, the ^3^H-labeled fatty acids were extracted and separated by thin layer chromatography (TLC). The plate was stained with iodine; the fatty acid “spot” was scraped off the plate, and the isolated fatty acid was added to scintillation fluid and counted in a liquid scintillation counter. De novo lipogenesis was shown as CPM of ^3^H_2_O incorporated into fatty acid /h/g liver protein.

### Lipid extraction and TG and CHOL mass measurement from cell, tissue and plasma

Liver lipid extraction and analysis were performed as previously described^39^. Briefly, approximately 300 mg of liver tissue was added to 20 volumes of 2:1 chloroform:methanol mixture and incubated for 24 h at room temperature. Following the incubation period, 0.2 volumes of 0.9% NaCl were added to the solvent mixture. The samples were thoroughly vortexed then centrifuged at 2000 rpm for 3 min. The upper aqueous phase was removed and the solvent layer was allowed to evaporate. The dried lipid was resuspended in 1 ml of 100% ethanol. Cell Lipids were extracted using a hexane:isopropanol (3:2) solvent mixture as described in ref. 41. TG and CHOL concentrations were determined using an Enzymatic/GPO endpoint method (Pointe Scientific, Canton, MI) as per the manufacturer’s instructions. Lipid data are expressed in milligrams of lipid per gram of liver tissue.

### Plasma insulin measurement

Plasmas were collected from mice after 12 hour fasting or 12 hour fasting followed by 6 hour refeeding. Plasma insulin contents were determined using an ultrasensitive mouse insulin ELISA kit (Crystal Chem, IL, USA) as per the manufacturer’s instructions.

### RNA isolation and qRT-PCR

Total RNA was isolated from tissues and cells using TRIzol (Life Technologies, Grand Island, NY). First strand cDNA was synthesized with oligo (dT) and random primers using a High-Capacity cDNA Reverse Transcription Kit with RNase Inhibitor (Life Technologies). Q-RT-PCR reactions were carried out using SYBR Green PCR Master Mix (Applied Biosystems, Life Technologies). Relative quantities of mRNA were calculated from threshold cycle (C_T_) values with the comparative C_T_ method, using 18S rRNA as an internal reference. Primer sequences are provided in the supplementary materials.

### Statistical analyses

Data obtained by densitometry or fluorography were evaluated using one-way ANOVA (GraphPad Prism 5, La Jolla, CA, USA). Post-test analysis was performed to determine the significance between groups, using unpaired two-way Student *t*-tests. All results are presented as means ± SEM. Asterisks (* or **) indicate statistically significant differences of *P* < 0.05 or *P* < 0.01, respectively, compared to controls.

## Additional Information

**How to cite this article**: Wang, H. *et al*. Glucagon regulates hepatic lipid metabolism via cAMP and Insig-2 signaling: implication for the pathogenesis of hypertriglyceridemia and hepatic steatosis. *Sci. Rep*. **6**, 32246; doi: 10.1038/srep32246 (2016).

## Supplementary Material

Supplementary Information
