# Supplementary material for: Glucagon regulates hepatic lipid metabolism via cAMP and Insig-2 signaling: implication for the pathogenesis of hypertriglyceridemia and hepatic steatosis
Source: Sci Rep. 2016 Sep 1;6:32246. doi: 10.1038/srep32246 (PMC5007496; doi:10.1038/srep32246)
Supplement: Supplementary Information [file srep32246-s1.pdf]

Supplemental Materials

Glucagon regulates hepatic lipid metabolism via cAMP and Insig-2 signaling: implication for the pathogenesis of hypertriglyceridemia and hepatic steatosis

Hai Wang[1‡], Miaoyun Zhao[1‡], Neetu Sud[1], Patricia Christian[1], Jing Shen[1], Yongyan Song[1], Anjeza Pashaj[1], Kezhong Zhang[2], Timothy Carr[1] and Qiaozhu Su[1*]

[1]The Department of Nutrition and Health Sciences, University of Nebraska-Lincoln, Lincoln, NE USA 68583-0806
[2]Wayne State University, School of Medicine, Detroit, Michigan, USA
[‡] These authors contributed equally to this work.

Materials and Methods

Primers used in this study

Table 1 Primers Used in This Study

18s	Forward	GATCCGAGGGCCTCACTAAAC	Mouse
	Reverse	TAAGTCCCTGCCCTTTGTACACA	
Insig-1	Forward	ACACGTGGGACCTAACT	Mouse
	Reverse	TCTGAAATGACCCGAGA	
Insig-2a	Forward	CCCTCAATGAATGTACTGAAGGATT	Mouse
	Reverse	TGTGAAGTGAAGCAGACCAATGT	
Insig-2b	Forward	CCGGGCAGAGCTCAGGAT	Mouse
	Reverse	GAAGCAGACCAATGTTTCAATGG	
SREBP-1c	Forward	CCAGCCTTTGAGGATAACCA	Rat
	Reverse	TGCAGGTCAGACACAGGAAG	
SREBP-2	Forward	CAGGGGTCTTCAGCATGATT	Rat
	Reverse	GGCAAGAGACCTGAGTCCTG	
FASN	Forward	TGGGTTCTAGCCAGCAGAGT	Mouse
	Reverse	ACCACCAGAGACCGTTATGC	
ACC	Forward	AGGCAGCAGTTGAAACCCTA	Mouse
	Reverse	TGAGCCTTGGTGTCTTCTCC	
SCD1	Forward	GAGAAGGGCGGAAAACTGGA	Mouse

	Reverse	TGAAGCACATCAGCAGGAGG	
LDLR	Forward	GGGAACATTTCGGGGTCTGT	Mouse
	Reverse	AGTCTTCTGCTGCAACTCCG	
HMGCR	Forward	AGCTTGCCCGAATTGTATGTG	Mouse
	Reverse	TCTGTTGTGAACCATGTGACTTC	
HMGCS	Forward	CCCTCCCTGGGAGATAAAGTG	Mouse
	Reverse	CCCGTGAATTGAAGCTGTCA	
Insig-2a	Forward	CGGTAGGGGGTACGGG	Human
	Reverse	CTAGATGTCTGTCAATGCAGGGTA	
Insig-1	Forward	CATGCGCTGCATAGCAGTTT	Human
	Reverse	GGCCACTTCTGGAACGATCA	

Supplementary Figures and Figure Legends:

Supplementary Figure 1

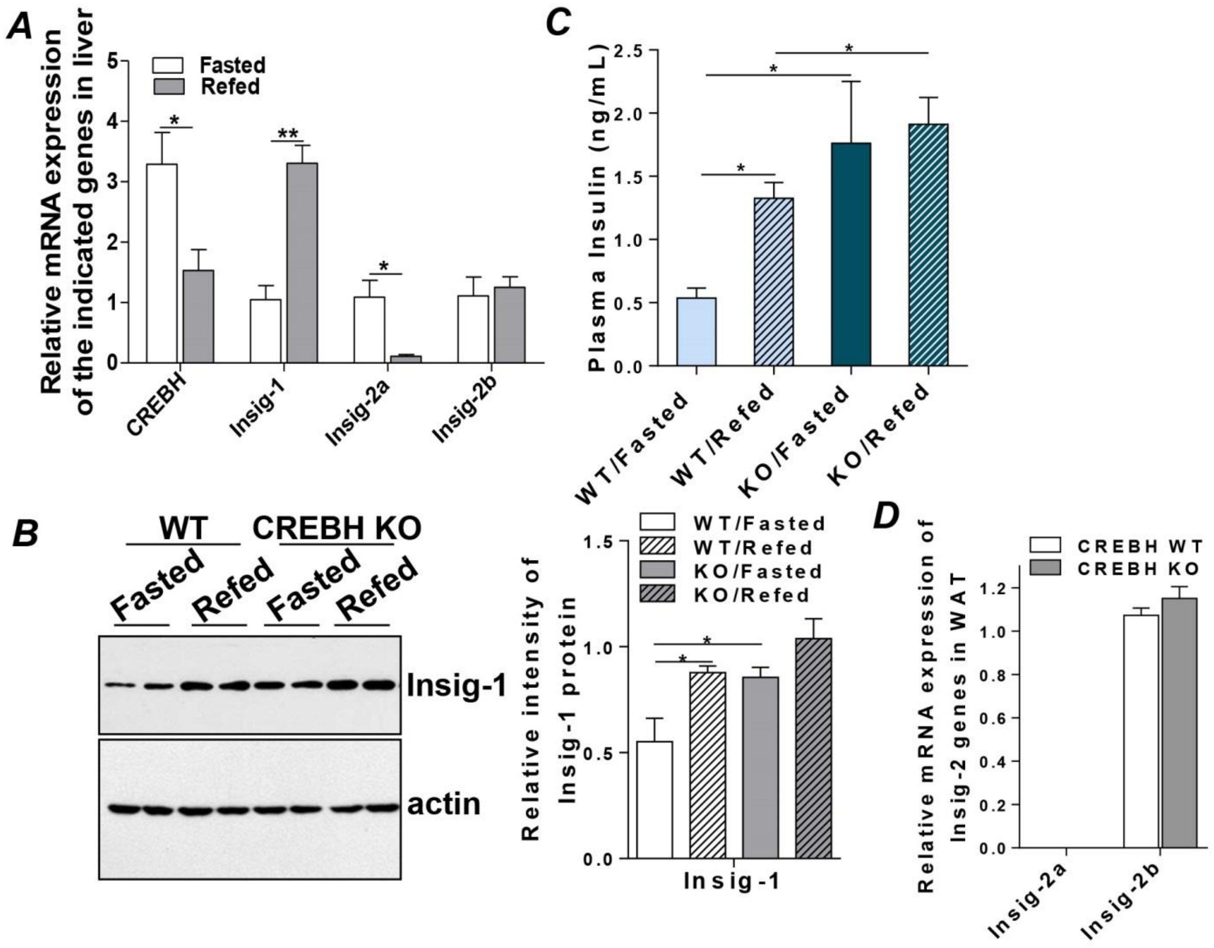

Supplementary Figure 1. (**A**) C57/B6 WT mice were divided into two groups (n=6/group) and subjected to the fasting and refeeding protocol as described in the Methods. Livers were then collected and used to analyze the relative mRNA levels of hepatic CREBH, Insig-1, Insig-2a, and Insig-2b by qRT-PCR. (**B** and **C**) CREBH-KO (KO) and control littermates were divided into fasted and refed groups and subjected to the fasting and refeeding protocol as described in the Methods. Plasmas and livers were then harvested for the following assays. (**B**) Immunoblot analysis of Insig-1 in the liver homogenates. (**C**) Plasma insulin contents. (**D**) White adipose tissues from WT and CREBH-KO mice were used to extract total RNA to analyze the mRNA expression of Insig-2a and Insig-2b. Data are expressed as means ± SEM (n = 3-6/group). *P < 0.05, **P<0.01 versus controls.

Supplementary Figure 2

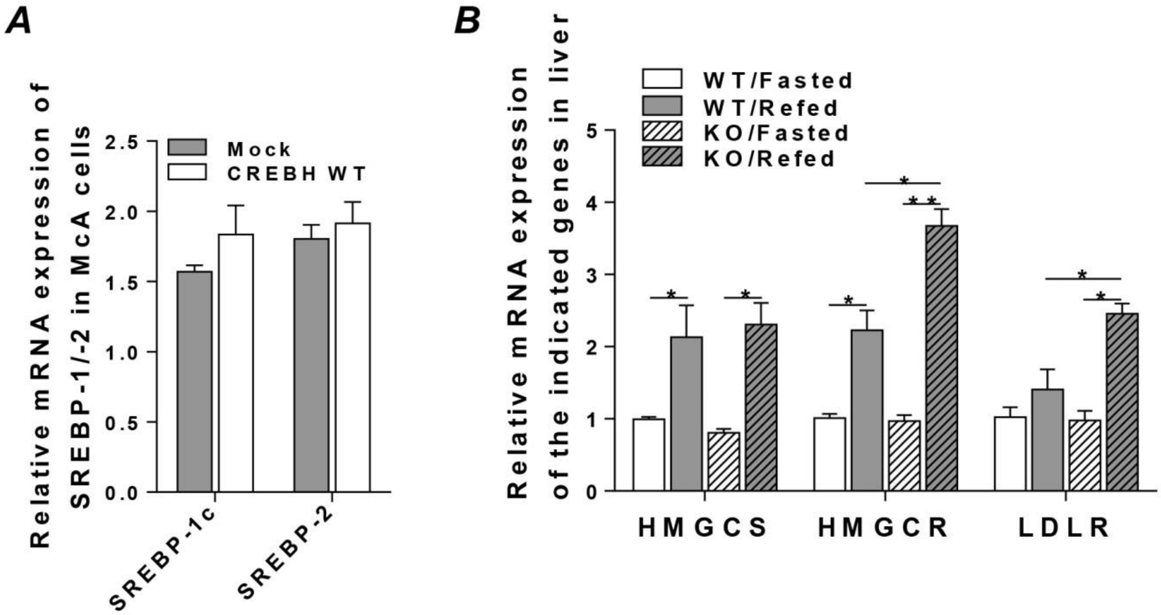

Supplementary Figure 2. (**A**) McA cells ($1x10^6$) were transfected with 1.5 μg of an empty vector (Mock) or 1.5 μg of a pCMV7.1 plasmid containing 3Flag-CREBH WT cDNA. After 48 hours, transfected cells were harvested for total RNA preparation. Relative mRNA expression of SREBP-1c and SREBP-2 were detected by qRT-PCR. (**B**) Relative mRNA expressions of SREBP-2 target genes, HMGCS, HMGCR, and LDLR, in the livers of WT and CREBH-KO (KO) mice after subjected to the fasted and refed protocol as described in the Methods, were determined by qRT-PCR. For animal studies, results are shown as mean ± SEM. n=6/group. For in vitro studies, data represent three experiments. *P < 0.05, **P < 0.01 versus controls.

Supplementary Figure 3

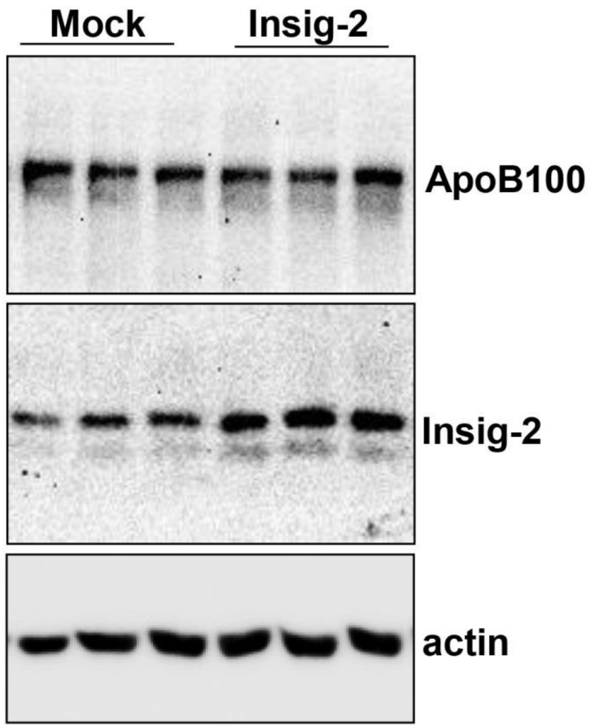

Supplementary Figure 3. VLDL-apoB in the culture media of McA cells transfected with a mock empty vector or pCMV-Insig-2 for 48 hours was determined by immunoblotting analysis (upper panel). Expression of Insig-2 in McA cells was also determined by immunoblotting analysis (middle panel). Results are shown as means ± SD for two experiments that were performed in triplicate.

Supplementary Figure 4

A

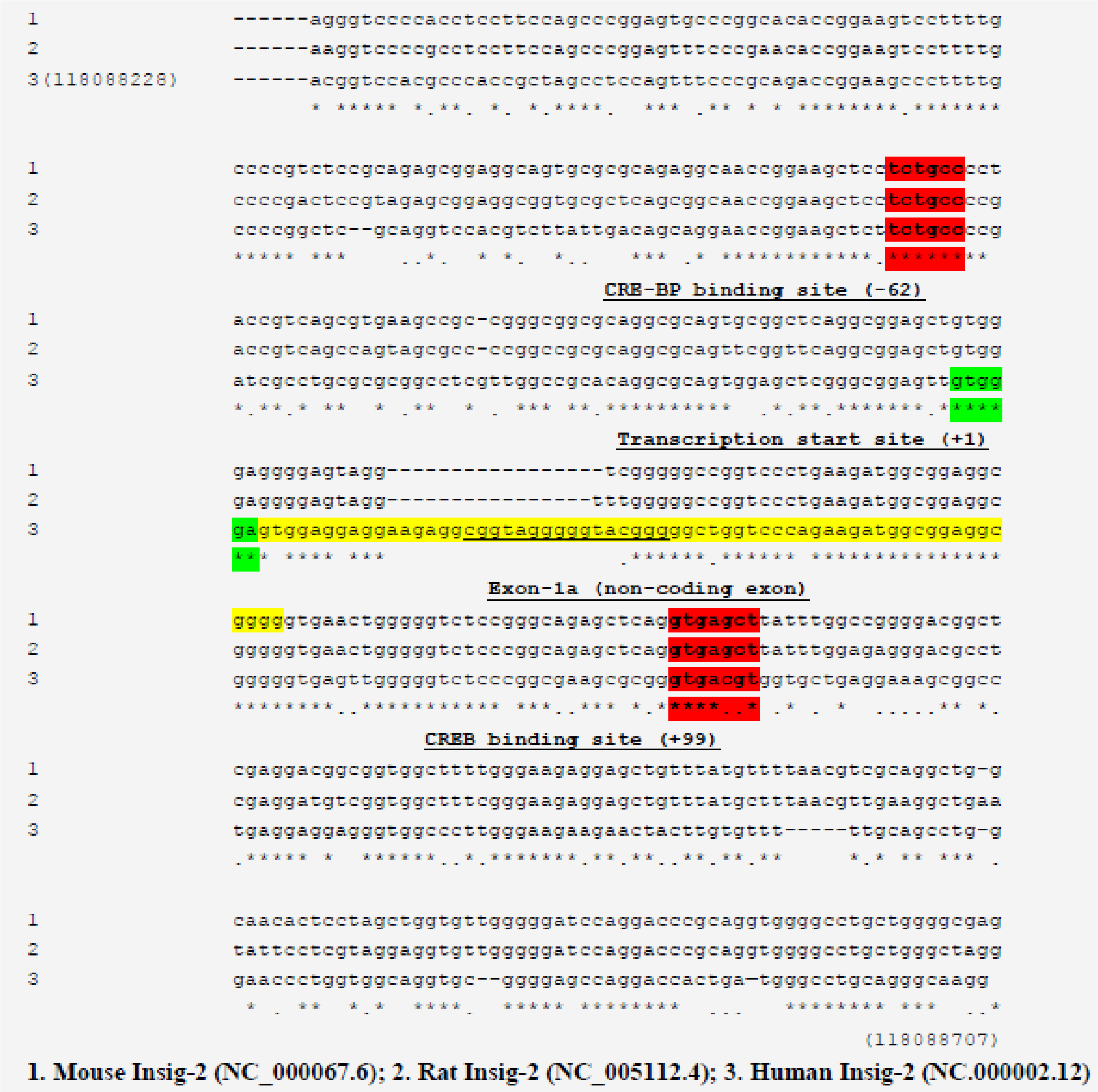

B

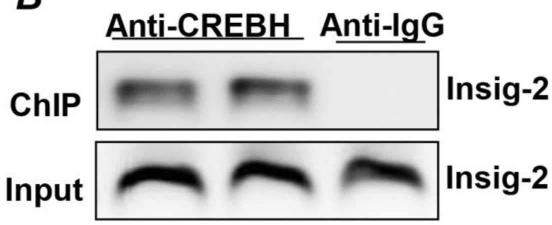

Supplementary Figure 4. Sequence alignment of mouse, rat and human Insig-2 promoter regions (**A**) The sequence alignment of the Insig-2 promoter regions (479 bps) of mouse, rat and human are presented to show the overall homology among these three species. Human Insig-2 (Insig-2a) was used as a prototype to indicate the transcriptional start site (green), the CRE-BP (-62) and CREB (+99) binding motifs (red), and the non-coding exon-1a (yellow). Conserved nucleotides among the three species are indicated with *. (**B**) ChIP assay of rat Insig-2 gene promoter in McA cells ($4x10^7$). Briefly, after cross-linking chromatin DNA to the interacting proteins, specific immunoprecipitation with an anti-CREBH antibody, or pre-immune IgG as a negative control, was performed as described in the Methods. PCR products of the Insig-2 promoter were analyzed after PCR amplification (36 cycles) by using a pair of primers flanking the CRE-BP binding elements (upper panel). Aliquots of 2% input DNA samples were used in PCR analysis as positive controls (lower panel).

Supplementary Figure 5

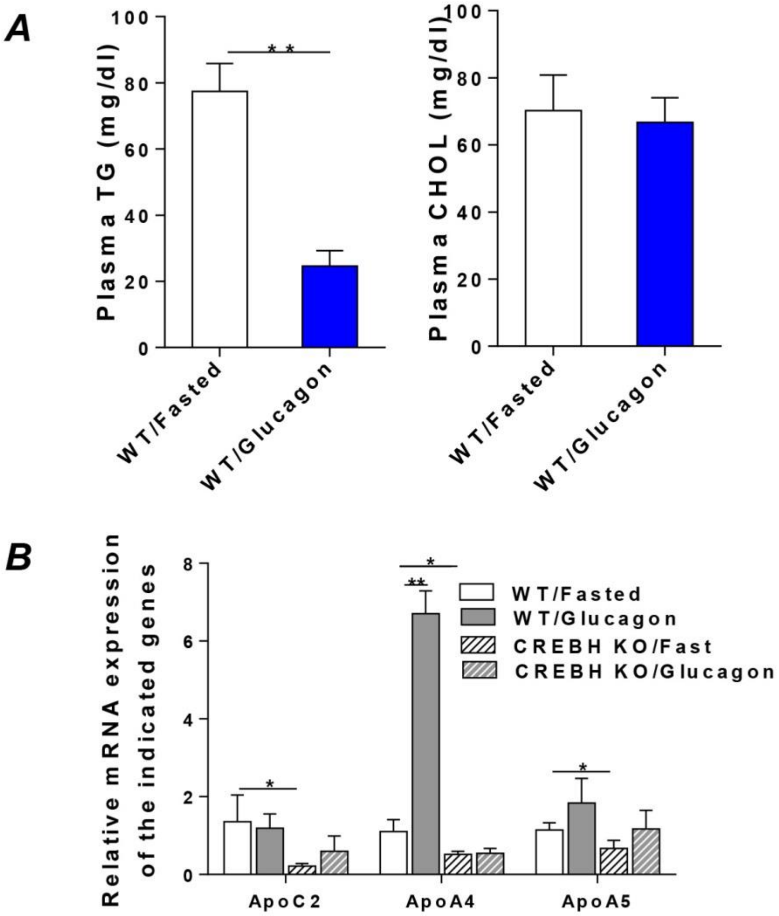

Supplementary Figure 5. (**A**) Plasma TG and CHOL in the WT mice after treated with glucagon as described in the Methods. (B) WT or CREBH-KO mice were untreated or treated with glucagon (30μg/kg) for 4 h as detailed in the Methods. Total RNA was extracted from liver tissues and used to determine the mRNA expression of apoC2, apoA4 and apoA5 by qRT-PCR. Results are shown as mean ± SEM. n=5-6/group. *P<0.05, **P < 0.01 versus controls.

Supplementary Figure 6

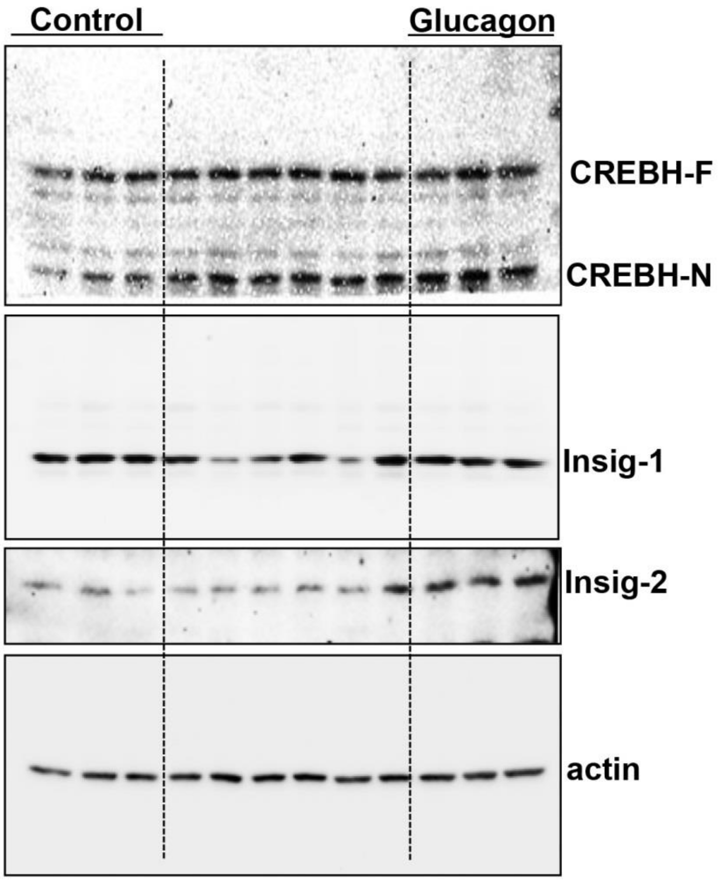

Supplementary Figure 6. Uncropped images for Fig. 5A. The same extracts were electrophoresed on PAGE gels and immunoblotted with the indicated antibodies. Fig. 5A was assembled from these images. Lanes in the middle are treatments not discussed in this manuscript.
